# A bioactive bovine whey protein extract improves intestinal barrier function in vitro

**DOI:** 10.3168/jdsc.2022-0245

**Published:** 2022-09-30

**Authors:** Dulantha Ulluwishewa, Jane Mullaney, Katharine Adam, Rod Claycomb, Rachel C. Anderson

**Affiliations:** 1 AgResearch Ltd., Te Ohu Rangahau Kai, Palmerston North 4474, New Zealand; 2 Riddet Institute, Massey University, Palmerston North 4442, New Zealand; 3 High-Value Nutrition National Science Challenge, Auckland 1142, New Zealand; 4 Quantec Ltd., Hamilton 3216, New Zealand

## Abstract

The human intestine plays an important role as a barrier against the ingress of pathogens and other harmful antigens. Accordingly, proper regulation of the intestinal barrier is essential for optimal health. Intestinal barrier function is regulated in part by the interactions between dietary compounds and the intestinal immune system. Bioactive whey proteins from bovine milk (such as lactoferrin, lactoperoxidase, and immunoglobulins) are known to exert a range of physiological functions, including modulation of the immune system, and thus have the potential to regulate intestinal barrier function. While the effects of individual whey proteins on intestinal barrier function have been studied to some extent, less is known about the potentially synergistic properties of whey protein mixtures. Here we investigated the effects of a bioactive bovine whey protein (BWP) extract containing all whey proteins with an isoelectric point >6.8 on intestinal barrier function in vitro. Intestinal epithelial cell (Caco-2) monolayers were treated with BWP before measuring the barrier integrity over 48 h by means of trans-epithelial electrical resistance (TEER). Treatment of epithelial monolayers with 1 mg/mL BWP resulted in an increase in TEER compared with untreated epithelial monolayers. To determine whether BWP could mitigate immune-mediated intestinal barrier dysfunction, we challenged differentiated Caco-2 cell monolayers with tumor necrosis factor α (TNFα) to obtain an in vitro model of a “leaky” intestinal epithelium. The TNFα challenge led to a decrease in TEER over time across untreated control monolayers, indicating a loss of barrier function. This loss of barrier function was mitigated in monolayers treated with 1 mg/mL BWP, but not monolayers treated with the equivalent amount of lactoferrin present in 1 mg/mL BWP. These data suggest that naturally co-occurring bioactive proteins together may enhance intestinal barrier integrity and protect against inflammation-induced barrier dysfunction to a greater extent than lactoferrin alone. Further work is required to determine the key proteins and protein combinations within BWP, and the mechanisms through which BWP modulates intestinal barrier function.

## Highlights

• We report on a whey extract containing all bovine milk whey proteins with pI >6.8.
• The whey extract improved Caco-2 cell transepithelial electrical resistance.
• The whey extract mitigated immune-mediated intestinal barrier dysfunction in vitro.

## Graphical Abstract

**Figure:**
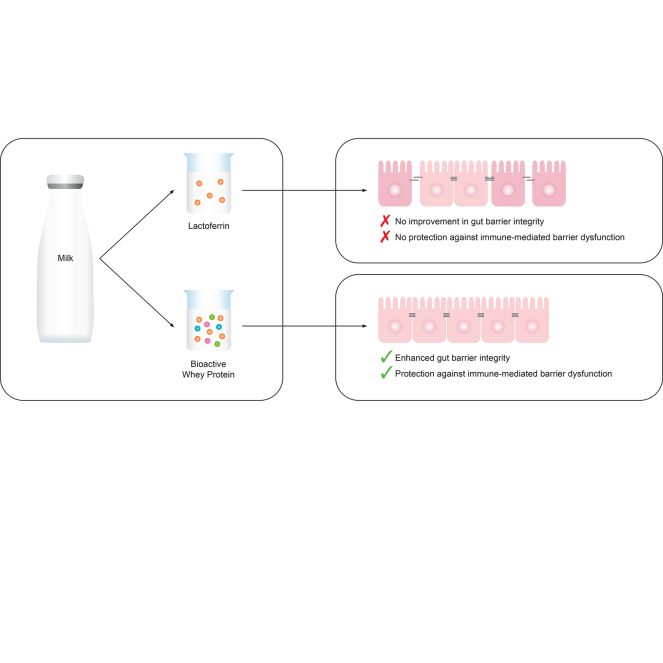
**Summary:** The human intestinal barrier plays an important role in health and disease. Individual whey proteins, such as lactoferrin, that are separated out from the whey, have been shown to improve intestinal barrier function. We hypothesized that the combination of whey proteins that naturally co-occur in milk could exert greater effects on intestinal barrier than lactoferrin alone. In this study we utilized a novel bioactive whey protein (BWP) extract containing all whey proteins in bovine milk with an isoelectric point (pI) >6.8. In support of the hypothesis, our data showed that the BWP extract, but not lactoferrin, (1) enhanced barrier integrity in healthy intestinal epithelial monolayers in vitro, and (2) protected against immune-mediated barrier dysfunction in an in vitro model of the intestinal epithelium. Future studies that consider the natural co-occurrence of bioactive proteins and their synergistic properties could aid in the development of improved dairy-based solutions for health.

The human intestine functions as a barrier between the external environment and the internal milieu, selectively allowing the absorption of nutrients, while preventing the influx of unwanted luminal antigens (Bischoff et al., 2014). Disruption of the intestinal barrier and increased permeability can lead to the passage of unwanted luminal antigens via the intestinal epithelium, which can trigger a local immune response via the activation of immune cells (e.g., lymphocytes, monocytes, and macrophages). This can ultimately result in an inflammatory cascade that exacerbates the loss of barrier function, and if left unmanaged, can lead to poor digestive or systemic health conditions. Dysfunction of the intestinal barrier is thought to play a role in chronic intestinal conditions (such as celiac disease, inflammatory bowel disease, and irritable bowel syndrome), as well as pathological or inflammatory states such as obesity, diabetes, and rheumatoid arthritis (Khoshbin and Camilleri, 2020). Even in healthy individuals, barrier dysfunction can be caused under environmental stress including hypoxia, hyperthermia, oxidative stress, the consumption of nonsteroidal anti-inflammatory drugs, or infection with pathogenic bacteria (Lambert, 2008; Chelakkot et al., 2018).

Diet is a major regulatory factor for intestinal barrier function, and the cross-talk between dietary components and the immune system plays a key role in the modulation of intestinal permeability (Farré et al., 2020). With consumers now seeking functional foods that are anti-inflammatory and support digestive and gut health (Sloan, 2020, 2021), there is growing interest in dietary compounds that can improve intestinal barrier integrity. Milk proteins, being a rich source of bioactive compounds, have been extensively studied and used for the production of health-promoting functional foods (Korhonen, 2009). In particular, the minor proteins that naturally occur in the whey fraction, such as immunoglobulins, lactoferrin, lactoperoxidase, and growth factors, have received considerable commercial and scientific interest (Korhonen, 2010). For example, lactoferrin supplements are widely considered beneficial due to their antimicrobial, antioxidative, and immunomodulatory properties (Superti, 2020). Notably, lactoferrin from bovine and other sources has also been shown to improve intestinal barrier function in vitro and in vivo (Hirotani et al., 2008; Wu et al., 2014; Zong et al., 2016; Hering et al., 2017; Zhao et al., 2019).

Despite several studies having evaluated the protective roles of specific peptides (such as lactoferrin) on intestinal barrier function, less is known about the potential synergistic properties of the protein milieu from whey. In this study we aimed to investigate the intestinal barrier enhancing potential of a commercially available bioactive whey protein (**BWP**) extract that contains all bovine milk whey proteins with an isoelectric point >6.8, present in same ratio as milk. Whey protein consists mainly of β-LG (∼50%) and α-LA (∼20%), but also contains minor proteins with potent bioactivity at a much lower concentration (Smithers, 2008). Separation of whey proteins based on an isoelectric point of 6.8 leads to the increased concentration of several minor bioactive whey proteins, by eliminating the major whey proteins such as α-LA and β-LG (Abd El-Salam and El-Shibiny, 2017).

The BWP extract used in this study is commercially produced via a process involving cationic chromatography and subsequent concentration of the eluate by ultrafiltration as depicted in Figure 1. Briefly, raw whole milk is defatted by centrifugation, and the resulting skim milk is processed through an ion exchange resin. Here several major milk components including lactose, casein, α-LA, β-LG, as well as all other whey proteins with an isoelectric point less than 6.8, are rinsed from the resin. The adsorbed whey proteins are subsequently eluted from the resin using 2 *M* sodium chloride to achieve a protein fraction containing whey proteins with an isoelectric point greater than 6.8. Finally, the eluted fraction is desalted, sterile filtered, and freeze-dried, before being milled and blended into a powder. By use of a proprietary analytical method relying on liquid chromatography tandem mass spectrometry with multiple reaction monitoring, the BWP extract was shown to contain lactoferrin (∼40% wt/wt), lactoperoxidase (∼18% wt/wt), angiogenin (∼10% wt/wt), immunoglobulin heavy chain (∼3% wt/wt), ribonuclease-4 (∼3% wt/wt), sulfhydryl oxidase (∼0.5% wt/wt), and lysosomal α-mannosidase (∼0.2% wt/wt). In addition, over 50 other minor proteins have been identified in the commercial BWP product (∼25% wt/wt).

**Figure 1:**
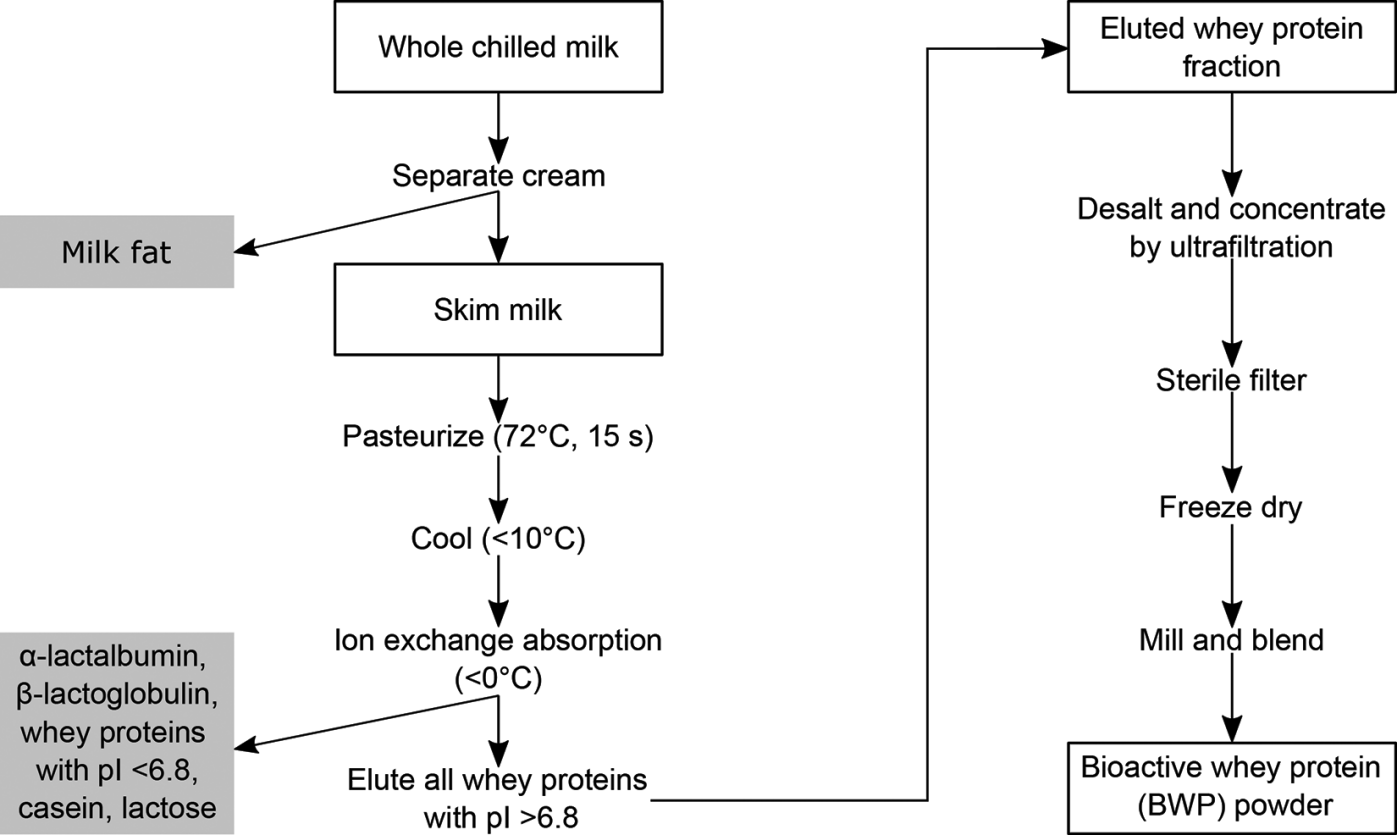
Flowchart of milk processing steps for production of bioactive whey protein (BWP) powder. A protein fraction containing all whey proteins in bovine milk with an isoelectric point (pI) greater than 6.8 was obtained utilizing cationic ion exchange chromatography. The protein fraction was desalted, sterilized, and freeze-dried, following which it was milled and blended to obtain BWP powder.

To investigate the effects of BWP on intestinal barrier function, we used a human colorectal adenocarcinoma cell line (Caco-2) as a model of the intestinal epithelium. Caco-2 cells are an appropriate model to assess the effects of milk proteins on the small intestine because these cells form a confluent monolayer in culture and spontaneously differentiate into polarized intestine enterocyte-like cells, characterized by an apical brush border and tight junctions between adjacent cells (Pinto et al., 1983; Hidalgo et al., 1989). Caco-2 cells (HTB37) were obtained from the American Type Culture Collection at passage 18 and used in experiments at passages 28 to 30. Caco-2 cells were grown in Gibco Minimum Essential Medium (**MEM**; Thermo Fisher Scientific) containing 10% (vol/vol) fetal bovine serum (FBS; Moregate BioTech) and 1% (vol/vol) MEM NEAA (100 × solution; NEAA; Thermo Fisher Scientific) at 37°C in a 5% CO_2_ humidified environment.

First, we carried out the WST-1 {4-[3-(4-iodophenyl)-2-(4-nitro-phenyl)-2H-5-tetrazolio]-1,3-benzene sulfonate} assay to determine the cytotoxic effects of BWP on Caco-2 cells. In principle, the WST-1 tetrazolium salt reacts with the mitochondrial succinate-tetrazolium reductase in viable Caco-2 cells to form a formazan dye which can be quantified by spectrophotometry. For these experiments, Caco-2 cells were seeded in 96-well flat-bottom plates at a density of 2 × 10^4^ cells/well and incubated at 37°C in 5% CO_2_. Twenty-four hours postseeding, the cell culture medium was replaced with treatment (0, 0.1, 0.5, 1.0, or 2.0 mg/mL BWP). Treatments were prepared by first dissolving BWP in MEM with 1% NEAA at a concentration of 5 mg/mL to obtain a stock solution. The stock solution was filter-sterilized using a 0.22-µm polyethersulfone membrane, following which treatments were prepared by diluting the stock solution to the desired concentration in MEM with 1% NEAA. All cells were cultured in the presence of treatment for 24 h at 37°C in a 5% CO_2_ atmosphere. Following treatment, the cell culture medium was replaced with 110 µL of WST-1 reagent (Roche) diluted 1:10 in MEM with 1% NEAA and incubated at 37°C for 1 h before measuring the absorbance of the formazan product at 450 nm using a Flexstation 3 spectrophotometer (Molecular Devices). Six replicate values were measured for each treatment. Background absorbance was measured at 600 nm and subtracted from the 450 nm measurements, and the corrected values were normalized by dividing by the average corrected value for the untreated (0 mg/mL BWP) control samples. A one-way ANOVA followed by the Dunnet post-hoc analysis was performed using the Prism software package (version 9.0.0, GraphPad Software) to compare the treatments against the control. The results indicated that that BWP at 2 mg/mL, but not 1 mg/mL, decreased cell viability (Figure 2a).

**Figure 2:**
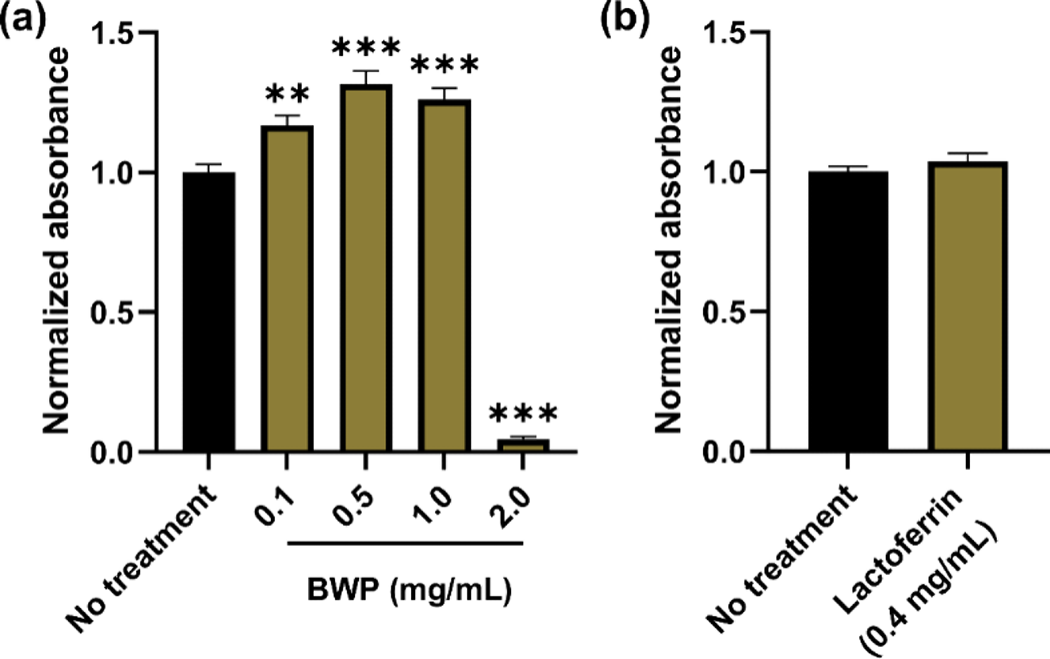
Cytotoxicity analysis of bioactive whey protein (BWP; a) and lactoferrin (b) on Caco-2 cells. Following exposure to treatments for 24 h, Caco-2 cells were incubated with WST-1 reagent for 1 h. The absorbance of the formazan dye produced by metabolically active cells was normalized by adjusting the untreated control cells to 1. The graph shows the mean (± SEM) normalized absorbance (n = 6–8). **P < 0.01, ***P < 0.0001 compared with no treatment control.

A maximum BWP concentration of 1 mg/mL was chosen for trans-epithelial electrical resistance (**TEER**) assay experiments based on the WST-1 assay (Figure 2a). Concentrations of 0.5 and 0.1 mg/mL BWP were also used to determine whether the proteins have a dose-dependent effect on barrier integrity. As previous studies have shown that lactoferrin can confer protective effects on the intestinal barrier (Hirotani et al., 2008; Hering et al., 2017; Zhao et al., 2019), we also used lactoferrin at 0.4 mg/mL (equivalent to the amount of lactoferrin in 1 mg/mL BWP) as a treatment to allow us to determine whether the observed effects of TEER could solely be due to the presence of lactoferrin in the BWP. To prepare the lactoferrin treatment, a 4 mg/mL stock solution was first prepared by dissolving lactoferrin (Bega-Bio freeze-dried lactoferrin, 96% purity; Tatura Milk Industries) in MEM with 1% NEAA. Following filter-sterilization of the stock solution using a 0.22-µm polyethersulfone membrane, the stock solution was diluted to 0.4 mg/mL in MEM with 1% NEAA. The WST-1 assay was repeated to ensure that 0.4 mg/mL lactoferrin did not exert cytotoxic effects on Caco-2 cells (Figure 2b). Eight replicate values were measured for each treatment. No difference could be detected between the lactoferrin-treated and control cells following analysis using a 2-tailed unpaired *t*-test in the Prism software package.

The effects of BWP on barrier integrity were then measured using TEER, which is an inverse measure of ion permeability across the epithelium, so it reflects the “tightness” of the barrier (Srinivasan et al., 2015). For this assay, Caco-2 cells were seeded on Transwell polyester filter inserts (6.5 mm, 0.4 µm pore; Corning) at a density of 8 × 10^4^ cells per insert. All experiments were undertaken 15 d postseeding to allow cells to differentiate. On the day before treatment, the apical medium from the Transwell inserts was replaced with MEM containing 1% (vol/vol) NEAA. The FBS was not included in the apical medium in case it interfered with the bioactivity of the treatments. The cells were allowed to equilibrate for approximately 24 h to ensure any treatment effects observed were not due to the removal of FBS.

On the day of treatment, the baseline (0 h) resistances across the cell monolayer was measured using Cell Culture Cup Chambers (EndOhm24, World Precision Instruments) and an Epithelial Volt/Ohm Meter (EVOM2, World Precision Instruments). For each monolayer, the net resistance was calculated by deducting the resistance of a blank Transwell membrane from the resistance measured. The TEER of the monolayer was calculated by multiplying the net resistance by the surface area of the Transwell membrane. Only Caco-2 monolayers that had a baseline TEER greater than 700 Ω·cm^2^ were used in assays. Immediately following the baseline measurement, the apical medium from the Transwell inserts was replaced with 260 µL of treatment, and the basal medium was replaced with 810 µL of fresh medium [MEM supplemented with 10% (vol/vol) FBS, 1% (vol/vol) NEAA].

The TEER was measured every 12 h over a period of 48 h, and the percentage change in TEER at each time point relative to the baseline TEER was calculated. The experiment was carried out 3 times, with 3 to 4 replicates per treatment in each experimental run. The effect of treatment on change in TEER over time was compared using a mixed-model ANOVA to account for the fact that the same monolayers were measured over time. Models were fitted by the REML method using the nlme package (Pinheiro et al., 2020) in R (Pinheiro et al., 2020, R Core Team, 2020). The statistical model included the effect of treatment, time, and their interaction as fixed effects, and the Transwell inserts nested within blocks (where one run of an experiment was considered a block) as a random effect. Pairwise comparisons were applied using estimated marginal means using the emmeans package (Lenth, 2021). The false discovery rate (q) was applied to the tests of the marginal means, with differences considered significant when q < 0.05.

The TEER increased between 0 and 12 h (q < 0.05) for all treatment groups, including untreated monolayers (Figure 3a). Thus, this initial increase in TEER was likely a due to the addition of the fresh culture medium, rather than the effect of any treatment. In untreated monolayers and monolayers treated with 0.1 or 0.5 mg/mL BWP, or 0.4 mg/mL lactoferrin, the TEER trended back toward baseline levels following the peak at 12 h. Within these treatment groups, comparisons of change in TEER between 0 and 24 h, 0 and 36 h, and 0 and 48 h did not show any statistically significant difference (q > 0.05). In contrast, when treated with BWP at the higher concentration of 1 mg/mL, the TEER across Caco-2 monolayers remained ∼15% to 18% higher than baseline for the duration of the assay (q <0.05 for change in TEER between 0 and 24 h, 0 and 36 h, and 0 and 48 h). Moreover, Caco-2 monolayers treated with 1 mg/mL BWP had a higher TEER than that of untreated monolayers between 24 and 48 h. Caco-2 monolayers treated with the equivalent amount of lactoferrin present in the 1 mg/mL BWP treatment did not differ from the untreated control cells at any of the time points. This suggests that the observed barrier-enhancing properties of BWP on unchallenged monolayers are not purely due to the presence of lactoferrin in the BWP extract.

**Figure 3:**
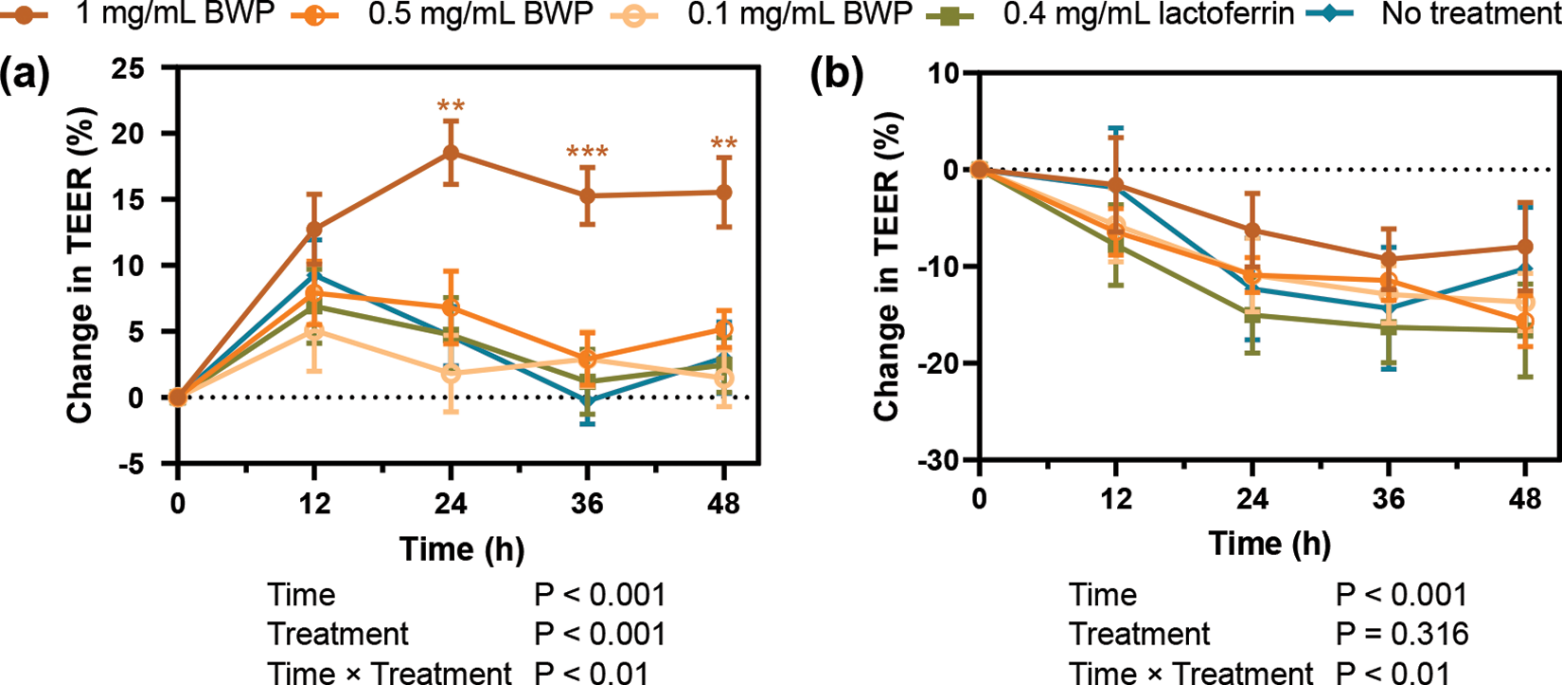
Effect of milk proteins on the trans-epithelial electrical resistance (TEER) across Caco-2 cell monolayers. (a) Graph shows mean (± SEM) change in TEER (n = 11; 3 experiments, 3–4 samples per treatment per experiment) across unchallenged Caco-2 monolayers. (b) Graph shows mean (± SEM) change in TEER (n = 9; 3 experiments, 3 samples per treatment per experiment) across Caco-2 monolayers challenged with TNFα. **q < 0.01, ***q < 0.001 compared with no treatment control.

We then sought to investigate whether the BWP extract could protect against inflammation-induced intestinal barrier dysfunction in vitro. To this end, we challenged the Caco-2 monolayers with TNFα, as this cytokine plays a critical role in barrier dysfunction in chronic intestinal conditions, while also being shown to induce comparable barrier defects in in vitro cell lines including Caco-2 (Hering et al., 2012). Experiments were carried out as described above; however, following the measurement of baseline TEER, the basal medium was replaced with 810 µL of fresh medium containing 100 ng/mL TNFα (Sigma-Aldrich). The experiment was carried out 3 times, with 3 replicates per treatment in each experimental run. As expected, TNFα challenge led to a decrease in TEER across untreated Caco-2 monolayers, indicating a loss of barrier function (Figure 3b). The TEER decreased ∼12% between 0 and 24 h (q < 0.001), and there was no further change between 24 and 36 h or 24 and 48 h. A similar trend was observed when monolayers were treated with BWP at a concentration of 0.1 or 0.5 mg/mL. In the latter treatment, the change in TEER decreased by a further ∼5% (relative to baseline TEER) between 24 and 48 h (q < 0.05). However, when Caco-2 monolayers were treated with a higher concentration of BWP (1 mg/mL), a statistically significant change in TEER was not observed between any of the time points, indicating that BWP is able to protect against TNFα-induced barrier dysfunction to some extent in Caco-2 monolayers. When TNFα-challenged Caco-2 monolayers were treated with an equivalent amount of lactoferrin present in the 1 mg/mL BWP treatment, the TEER decreased between 0 and 24 h (q < 0.001), and 24 and 48 h (q < 0.001), indicating the protective effects of BWP are not solely due to the lactoferrin component.

Because 1 mg/mL BWP resulted in an increase in TEER across Caco-2 monolayers between 24 and 48 h treatment relative to untreated cells, we sought to investigate the effect of BWP on genes known to be involved in epithelial barrier regulation, specifically, myosin light chain kinase (*MLCK1*), claudin 1 (*CLDN1*), tight junction protein 1 (*ZO-1*), and occludin (*OCLN*) (Cunningham and Turner, 2012; Chelakkot et al., 2018; Camilleri, 2019). Hence Caco-2 cell monolayers were treated with 0 (untreated), 0.5, or 1 mg/mL BWP for 36 h (n = 9; 3 experiments, 3 samples per treatment per experiment), and the gene expression level changes (relative to untreated cells) were determined using quantitative real-time PCR (**qPCR**). Following the treatment period, RNAprotect Cell Reagent (Qiagen) was added to the Transwell insert to lyse the Caco-2 cells and stabilize the RNA. Total RNA was extracted using an RNeasy mini kit (Qiagen) as per the manufacturer's protocol, and 1 μg of total RNA was reverse-transcribed to cDNA using SuperScript IV VILO Master Mix (Thermo Fisher Scientific). Expression levels of each gene were determined using pre-designed and pre-validated TaqMan Gene Expression Assays (Thermo Fisher Scientific) and TaqMan Fast Advanced Master Mix (Thermo Fisher Scientific). All qPCR reactions were completed as duplicate 20-µL reactions on a QuantStudio3 real-time thermal cycler (Thermo Fisher Scientific). Relative gene expression was calculated using the quantitative cycle (Cq) values as described by Taylor et al. (2019). For each target gene, the relative quantity was divided by the geometric mean of the relative quantities of 3 reference genes (*YWHAZ, SDHA*, and *GAPDH*) to obtain the normalized relative expression of the target gene per sample (Vandesompele et al., 2002). Statistical analysis was performed using log-transformed normalized relative expression per sample (Kitchen et al., 2010) using a one-way ANOVA followed by the Tukey's multiple comparisons test to compare the means of the treatments against the untreated control. Our analysis did not find differences in gene expression between untreated Caco-2 monolayers and those treated with BWP for 36 h (data not shown, *P* > 0.05). This suggests that the regulation of intestinal barrier function by BWP does not involve the modulation of expression of our target genes at the 36 h time point. However, whether gene expression changes occur at an earlier time point, or whether the BWP regulates barrier function via posttranscriptional modification is yet to be determined.

In summary, our data showed that BWP (1) enhances TEER across healthy (unchallenged) intestinal epithelial monolayers in vitro, and (2) protects against TNFα-induced reduction in TEER in an in vitro model of the intestinal epithelium. These data indicate that BWP may enhance intestinal barrier integrity and offer protection against inflammation-induced barrier dysfunction via its interactions with the intestinal epithelium. Although the BWP extract was primarily composed of lactoferrin, treatment of cells with solely lactoferrin could not confer barrier-enhancing and protective effects observed for BWP. This suggests that one or several of the other bioactive proteins present in BWP, or lactoferrin in combination with other proteins in BWP, may be responsible for the observed effects. Interestingly, we have shown in a previous study (Anderson et al., 2017) that lactoferrin isolated from milk collected at the start of the milking season (early-lactation), but not lactoferrin isolated from milk collected during the rest of the milking season (mid-lactation), was able to enhance TEER across unchallenged Caco-2 monolayers. We subsequently found that the early-lactation lactoferrin samples, but not the mid-lactation lactoferrin samples, contained angiogenin, which leads us to hypothesize that angiogenin, present at ∼10% (wt/wt) in BWP, may play a key role in the barrier promoting effects observed in the present study. However, further research is required to confirm the key components and synergistic properties of BWP involved in intestinal barrier regulation, and the mechanisms through which they interact with the interstitial epithelial cells. Our preliminary in vitro findings suggest that consumption of BWP may aid in digestive and gut health, and a detailed understanding of the barrier-enhancing and protective mechanisms of the whey protein milieu will aid in the development of novel functional foods.
